# Effect of smoking-related features and 731 immune cell phenotypes on esophageal cancer: a two-sample and mediated Mendelian randomized study

**DOI:** 10.3389/fimmu.2024.1336817

**Published:** 2024-03-27

**Authors:** Kaiqi Yang, Shaoya Li, Yuchen Ding, Xiaodie Meng, Changhao Zhang, Xiujing Sun

**Affiliations:** Department of Gastroenterology, Beijing Friendship Hospital, Capital Medical University, National Clinical Research Center for Digestive Disease, Beijing Digestive Disease Center, Beijing Key Laboratory for Precancerous Lesion of Digestive Disease, Beijing, China

**Keywords:** Mendelian randomization, smoking-related features, immune cell phenotypes, esophageal cancer, mediation effect

## Abstract

**Introduction:**

Numerous observational studies have indicated that smoking is a substantial risk factor for esophageal cancer. However, there is a shortage of research that delves into the specific causal relationship and potential mediators between the two. Our study aims to validate the correlation between smoking-related traits and esophageal cancer while exploring the possible mediating effects of immune factors.

**Methods:**

Initially, we conducted bidirectional univariate Mendelian Randomization (MR) analyses to forecast the causal effects linking smoking-related traits and esophageal cancer. Subsequently, we employed a two-step MR analysis to scrutinize immune cell phenotypes that could mediate these effects. Finally, the coefficient product method was employed to determine the precise mediating impact. Additionally, we have refined our sensitivity analysis to ensure the reliability of the outcomes.

**Results:**

After analysis, Smoking status: Never had a significant negative association with the incidence of esophageal cancer (inverse-variance weighted (IVW) method, p=1.82e-05, OR=0.10, 95%CI=0.04~0.29). Ever smoked (IVW, p=1.49e-02, OR=4.31, 95%CI=1.33~13.94) and Current tobacco smoking (IVW, p=1.49e-02, OR=4.31, 95%CI=1.33~13.94) showed the promoting effect on the pathogenesis of esophageal cancer. Through further examination, researchers discovered 21 immune cell phenotypes that have a causal relationship with esophageal cancer. After careful screening, two immune cell phenotypes were found to have potential mediating effects. In particular, it was observed that in the case of the preventive effect of Smoking status: Never on esophageal cancer, the absolute count of CD62L plasmacytoid dendritic cells mediated a reduction of 4.21%, while the mediating effect of CD27 in CD20-CD38-B cells was -4.12%. In addition, sensitivity analyses did not reveal significant heterogeneity or level pleiotropy.

**Conclusion:**

The study provides new evidence for the causal relationship between smoking-related features and esophageal cancer and proposes immune factors with potential mediating effects. However, this finding needs to be further demonstrated by more extensive clinical studies.

## 1 Introduction

Esophageal cancer is a prevalent type of cancer that has affected a staggering 604,100 individuals and led to 544,100 deaths globally in 2020 (1). The incidence of this cancer is increasing in Western countries. However, despite significant advancements in patient management and treatment, the overall survival rate after 5 years remains low, at approximately 10%. However, after esophageal cancer surgery, the survival rate slightly improves, ranging from 15-40% (2). To enhance the prognosis of this cancer, it is crucial to identify, explore, and intervene in all potential risk factors. Previous observational studies suggest that smoking (3), being white (4, 5), and gastroesophageal reflux disease (6, 7) are among the possible risk factors for esophageal cancer.

Research has demonstrated that smoking is a significant risk factor for esophageal cancer. According to studies, current smokers face a higher chance of developing esophageal adenocarcinoma compared to non-smokers (odds ratio (OR) = 1.96; 95% confidence interval (CI) = 1.64-2.34) (3). Even those who have quit smoking for a decade are more likely to develop esophageal adenocarcinoma than those who have never smoked (OR = 1.72; 95%CI, 1.38-2.15). Furthermore, continuing to smoke increases the risk of cancer in Barrett’s esophagus (8).

It’s common knowledge that tobacco smoke contains harmful substances like carbon monoxide and nicotine, which can trigger the production of various immune or inflammatory mediators in the body (9, 10). Recent research has shown that cigarette smoke exposure can cause changes in the immune system, including the increased release of IL-33 in epithelial cells and altered expression of the IL-33 homologous receptor ST2 in different immune cells (11). The immune system plays a vital role in preventing cancer by producing interferon (IFN)-γ and cytotoxins that can inhibit cancer progression. However, chronic inflammation caused by factors like smoking may override the effects of these cells and promote cancer progression (12–15). In addition, autoimmune diseases have been shown to support the development of many cancers due to ongoing immune system activity (16–18). Overall, smoking can hurt the immune system, promoting the occurrence and progression of esophageal cancer.

Mendelian randomization (MR) is a powerful epidemiological analysis method that predicts causal associations. This approach leverages genetic variation, such as single nucleotide polymorphisms (SNPs), as an instrumental variable (IV) to represent exposure factors. Since these SNPs are randomly distributed and independent of environmental factors and other confounders (19), MR design offers a rigorous explanation of causal relationships between complex factors.

In this study, we first evaluated the effect between multiple smoking-related features and susceptibility to esophageal cancer using univariate bidirectional MR analysis. We then used a two-step MR analysis to screen for immunophenotypes that could be potential mediators and their mediating effects between never-smoking and esophageal cancer.

## 2 Materials and methods

### 2.1 Study design

Our research employed genetic variants as instrumental variables for Mendelian randomization analysis. The credibility of our MR study is grounded in three key assumptions: (1) the correlation hypothesis, indicating a robust connection between genetic variation and exposure; (2) the independence hypothesis, affirming that genetic variation is not connected to any confounding variables that could impact the link between exposure and outcome; and (3) the Exclusion-Limit Hypothesis, which maintains that genetic variation influences outcomes solely through exposure (20).

### 2.2 Description of the data source

The data related to smoking encompasses smoking status (whether one has never smoked or is a current smoker) and smoking initiation. All of the relevant genetic information is sourced from public databases. The genetic association between esophageal cancer and genetics was derived from two separate GWAS data sets detailed in the table. All of the above data can be found in **Table 1**. Furthermore, the genetic data for 731 immune cell traits, identified as GCST0001391 to GCST0002121, were obtained from the GWAS Public Catalog. This comprehensive resource offers the most up-to-date report on genetic loci for immune cell traits, including absolute cell (AC) counts (n = 118), median fluorescence intensity (MFI) reflecting surface antigen levels (n = 389), morphological parameters [MP] (n = 32), and relative cell (RC) counts (n = 192) (21). The original GWAS on immune profiles was conducted using data from 3,757 European individuals with no overlapping cohorts. The study analyzed roughly 22 million SNPs genotyped with high-density arrays attributed to reference panels based on Sardinian sequences. Associations were tested after adjusting for covariates such as sex, age, and age2 (22). It is important to note that all data used in this study is at the GWAS abstract level. Therefore, ethical approval and informed consent were obtained in the original research.

**Table 1:** Details of the studies included in the Mendelian randomization analyses.

Phenotype	Consortium /Author	Ethnicity	Sample size	Year	Number of SNPs	Web source
Smoking status: Never	Neale lab	European	359,706	2018	13,586,591	https://gwas.mrcieu.ac.uk/datasets/ukb-d-20116_0/
Ever smoked	MRC-IEU	European	461,066	2018	9,851,867	https://gwas.mrcieu.ac.uk/datasets/ukb-b-20261/
Smoking initiation	GSCAN	European	607,291	2019	11,802,365	https://gwas.mrcieu.ac.uk/datasets/ieu-b-4877/
Current tobacco smoking	Neale Lab	European	337,030	2017	9,178,635	https://gwas.mrcieu.ac.uk/datasets/ukb-a-16/
Esophageal cancer	Sakaue S	European	476,306	2021	24,194,380	https://gwas.mrcieu.ac.uk/datasets/ebi-a-GCST90018841/

### 2.3 Selection of genetic instrumental variables

To ensure high-quality results, we implemented a rigorous quality control procedure to identify Type IV genes that align with the MR hypotheses. For Type IV genes related to smoking-related traits and esophageal cancer, we set the p-value threshold to p<5e-8, while for immune cell signature-related genes, it was p<5e-06. In the MVMR analysis, we used the same p-value threshold (p<5e-06) for both immune cell phenotype and smoking status to examine the mediated relationship. Our approach also involved applying a linkage imbalance clustering algorithm with R2<0.001 and a window size of 10,000 kb. To ensure that effector alleles are consistent, we harmonized the exposure and outcome datasets by removing SNPs with intermediate allele frequencies and ambiguous SNPs with inconsistent alleles. Lastly, we calculated the F-statistic for each SNP using the following equation to assess the strength of the IV:


$$F=\frac{N-K-1}{K}\times \frac{R^{2}}{1-R^{2}}$$

In this equation, F is equal to the numerator (N-K-1) divided by the denominator, which is ((K x R^2)/(1-R^2)). R^2 represents the proportion of variations explained by the IV (23). Since the F-statistic for all SNPs exceeded 10, only SNPs that undergo strict screening will be utilized as an IV for future analyses. N denotes the sample size of the exposed dataset, and K represents the number of SNPs.

### 2.4 Univariate MR analysis

To predict the impact of various smoking characteristics on esophageal cancer, three complementary methods were employed for univariate MR analysis: IVW, MR-Egger, and Weighted-median methods. IVW method was considered the primary causal estimation method, providing accurate results when all selected SNPs are valid IVs (24). MR-Egger method yields consistent causal estimates under the InSIDE assumption independent of the instrumental strength of direct influences, even if genetic IV is invalid. However, it is important to note that this method is imprecise and susceptible to peripheral genetic variation (25). The weighted median method, on the other hand, calculates the weighted median of Wald ratio estimates without InSIDE assumptions and is robust to horizontal pleiotropic bias. Compared with the MR-Egger method, the weighted median method has a lower type I error and a higher causal estimation power (26). A causal relationship between exposure and outcome was considered when the IVW analysis results were P<0.05, and the results of the three methods were consistent. For MR analysis between immune cell phenotype and esophageal cancer, the results were corrected for FDR, and a fixed P value meeting P<0.05 was considered indicative of a causal relationship.

### 2.5 Sensitivity analysis

Conducting sensitivity analysis to evaluate heterogeneity and potential pleiotropy that could significantly violate MR analysis requirements is crucial. Horizontal pleiotropy may occur when IVs impact outcomes through pathways other than exposure. To ensure the accuracy of the findings, we utilized several methods in this study. These methods included the Cochran Q test, the MR-Egger intercept test, and the MR-Pleiotropy RESidual Sum and Outliers (MR-PRESSO). In the presence of heterogeneity, a Cochran Q test result with p < 0.05 was deemed significant (27). The MR-Egger intercept was utilized to assess the offset due to the IV’s invalidity (28). Finally, we employed MR-PRESSO to re-examine the study for any potential horizontal pleiotropy (29).

### 2.6 Reverse MR analysis

Before further research, we validated the directionality of causal effects. We performed two-sample univariate MR analyses using esophageal cancer as an exposure factor and various smoking characteristics as outcomes. Based on the analysis results, we verify our judgment on the directionality of causal effects.

### 2.7 Mediated MR analysis

Our analysis focused on the impact of smoking status on the development of esophageal cancer. We utilized a two-step MR approach to investigate potential immunophenotypes that could act as mediators between never-smokers and those with esophageal cancer. The Two-step Mediation Regression (MR) technique can be likened to the coefficient product method. It involves calculating two MR estimates, namely, the causal effect of the exposure on the mediator, and the causal effect of the mediator on the outcome. By multiplying these two estimates, one can obtain an estimate of the indirect effect. First, we assessed the causal effects of 731 immunophenotypes on esophageal cancer development. Then, we analyzed the immunophenotype that had a causal effect on esophageal cancer to observe its impact on never-smokers. Our findings identified a positive immunophenotype that we consider a potential mediator. To further investigate, we estimated the overall effect of never smoking on the risk of esophageal cancer and its impact on potential intermediate mediators. We then evaluated the direct impact of potential intermediate mediators on esophageal cancer. Finally, we calculated the proportion of mediated effects. Our approach utilized univariate MR and the coefficient product method (30–32).

All statistical analysis and data visualization were performed using R programming software (R4.2.3), including the “TwoSampleMR” R package (Version 0.5.7) and the “MRPRESSO” (version 1.0) R package (PMID: 24114802). The “forestploter” R package (version 1.1.1) generates a forest plot. Use the “circlize” R package to create a Ring heatmap.

## 3 Results

### 3.1 Effect of smoking-related features on esophageal cancer

**Figure 1:**
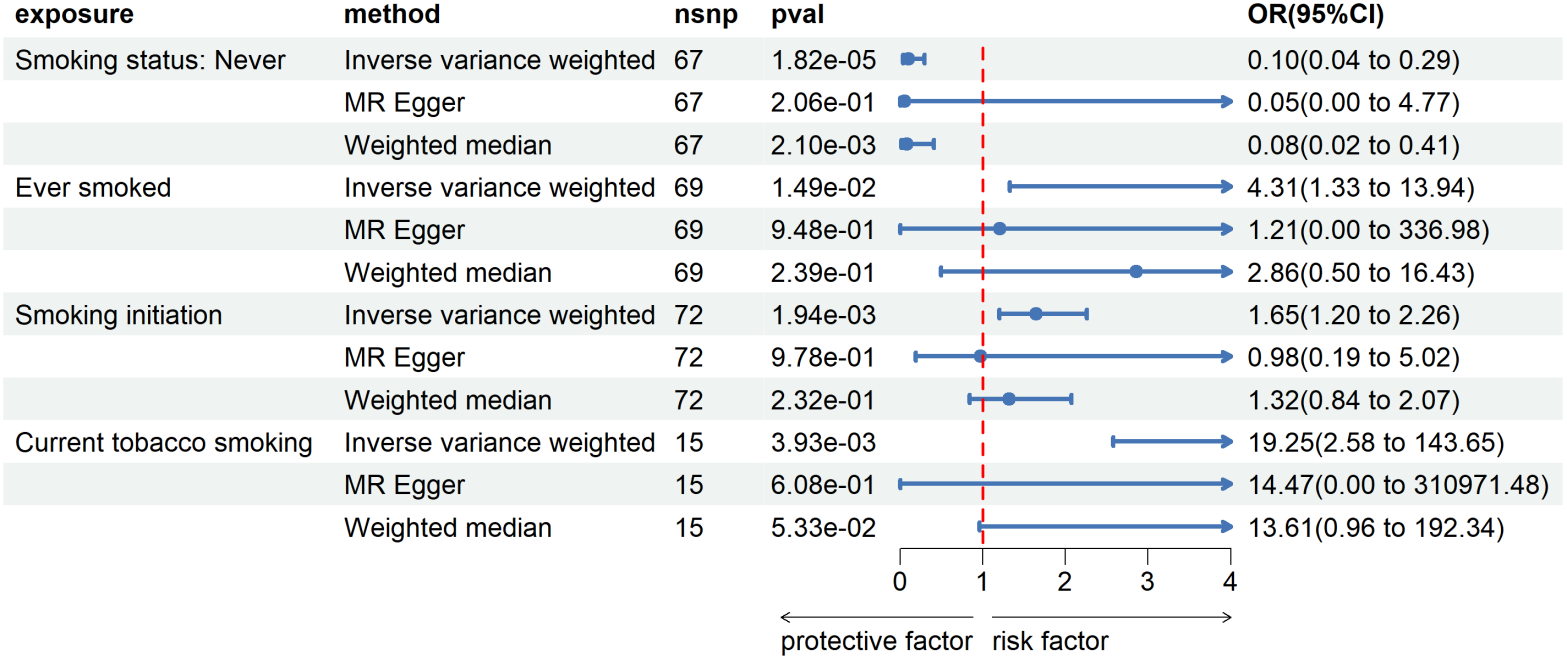
Forest plot of Mendelian randomization analyses of smoking-associated phenotypes on esophageal cancer. OR, odds ratio. 95%CI, 95% confidence interval. nsnp, number of single nucleotide polymorphisms.

According to the findings in **Figure 1**, the genetic prediction of never smoking was significantly and negatively linked to the incidence of esophageal cancer, as demonstrated by the two-sample univariate MR analysis (IVW method, p=1.82e-05, OR=0.10, 95%CI=0.04~0.29). In contrast, ever smoking (IVW, p=1.49e-02, OR=4.31, 95%CI=1.33~13.94) and current tobacco smoking (IVW, p=1.49e-02, OR=4.31, 95%CI=1.33~13.94) were associated with an increased risk of esophageal cancer. In the case of partial associations, both the weighted median MR and MR-Egger OR values were in agreement with the IVW method. However, the confidence intervals were wider owing to reduced statistical power (29). It’s worth noting that while the IVW method suggests that smoking initiation may promote the development of esophageal cancer, the MR Egger method indicates the opposite effect. After conducting sensitivity analysis, no significant heterogeneity or level of pleiotropy was detected, and all results can be found in **Supplementary Materials Table 1**. It is noteworthy that all IVs chosen in the studies mentioned above possessed F-statistic values exceeding 10 (please refer to **Supplementary Materials Tables 5–8**).

### 3.2 Effects of esophageal cancer on various smoking characteristics

**Figure 2:**
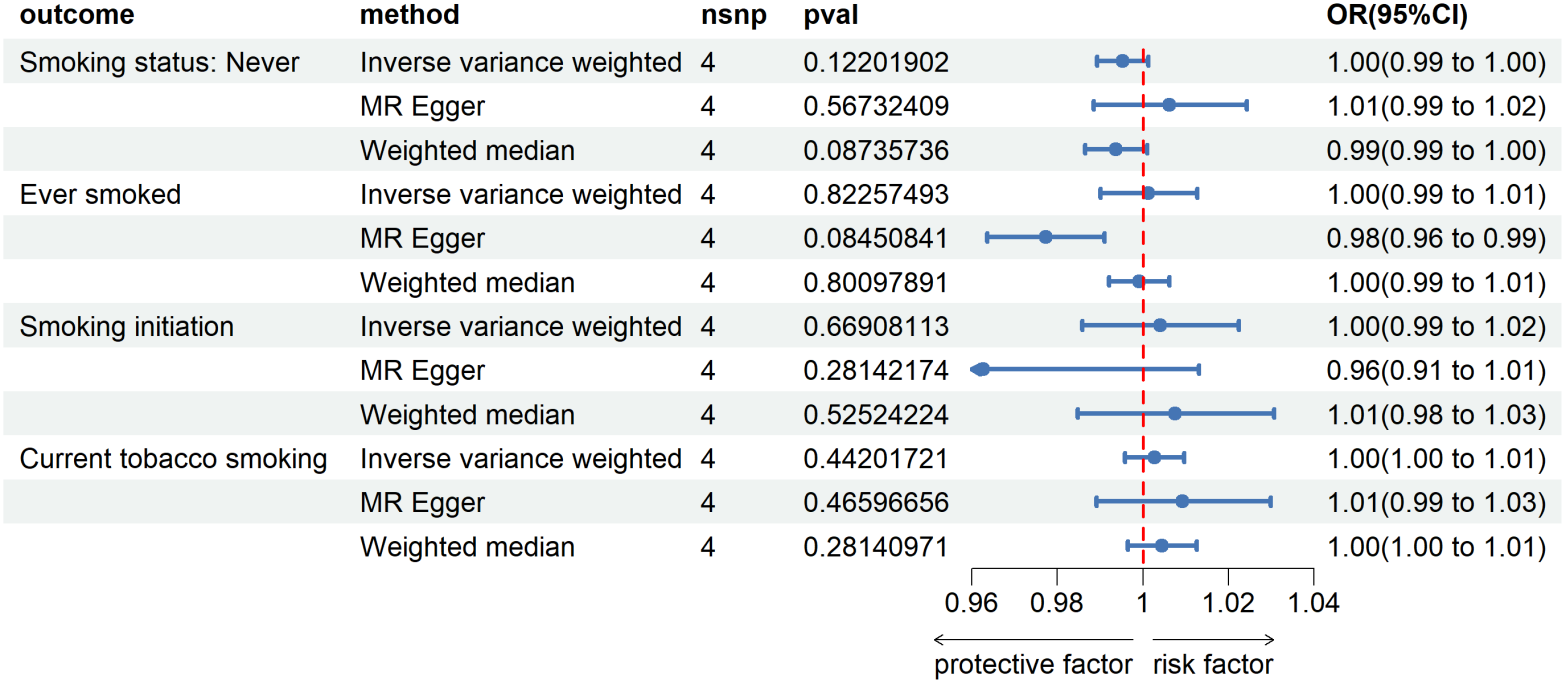
Forest plot of Mendelian randomization analyses of esophageal cancer on smoking-associated phenotypes. OR, odds ratio. 95%CI, 95% confidence interval. nsnp, number of single nucleotide polymorphisms.

After reversing MR analysis, as shown in **Figure 2**, compared to patients with non-esophageal cancer, the results showed that esophageal cancer had no significant effect on Smoking status: Never (IVW method, p=0.12, OR=1.00, 95%CI=0.99~1.00), which verified our conjecture about the directionality of the causal association between the two. For the remaining three smoking-related features, esophageal cancer was also found to have no promoting or inhibitory effect on them. We then refined the sensitivity analysis, and the results are presented in the **Supplementary Materials Table 2**. Notably, genetic prediction of the development of esophageal cancer on Ever smoked showed significant heterogeneity and level pleiotropy (IVW method, Q_pval=1.88e-3; MR PRESSO, Global Test_pval=0.032), suggesting that the causal relationship between the two should be carefully considered and verified in more complete large-scale GWAS data.

### 3.3 Effect of immune cell phenotypes on esophageal cancer

**Figure 3:**
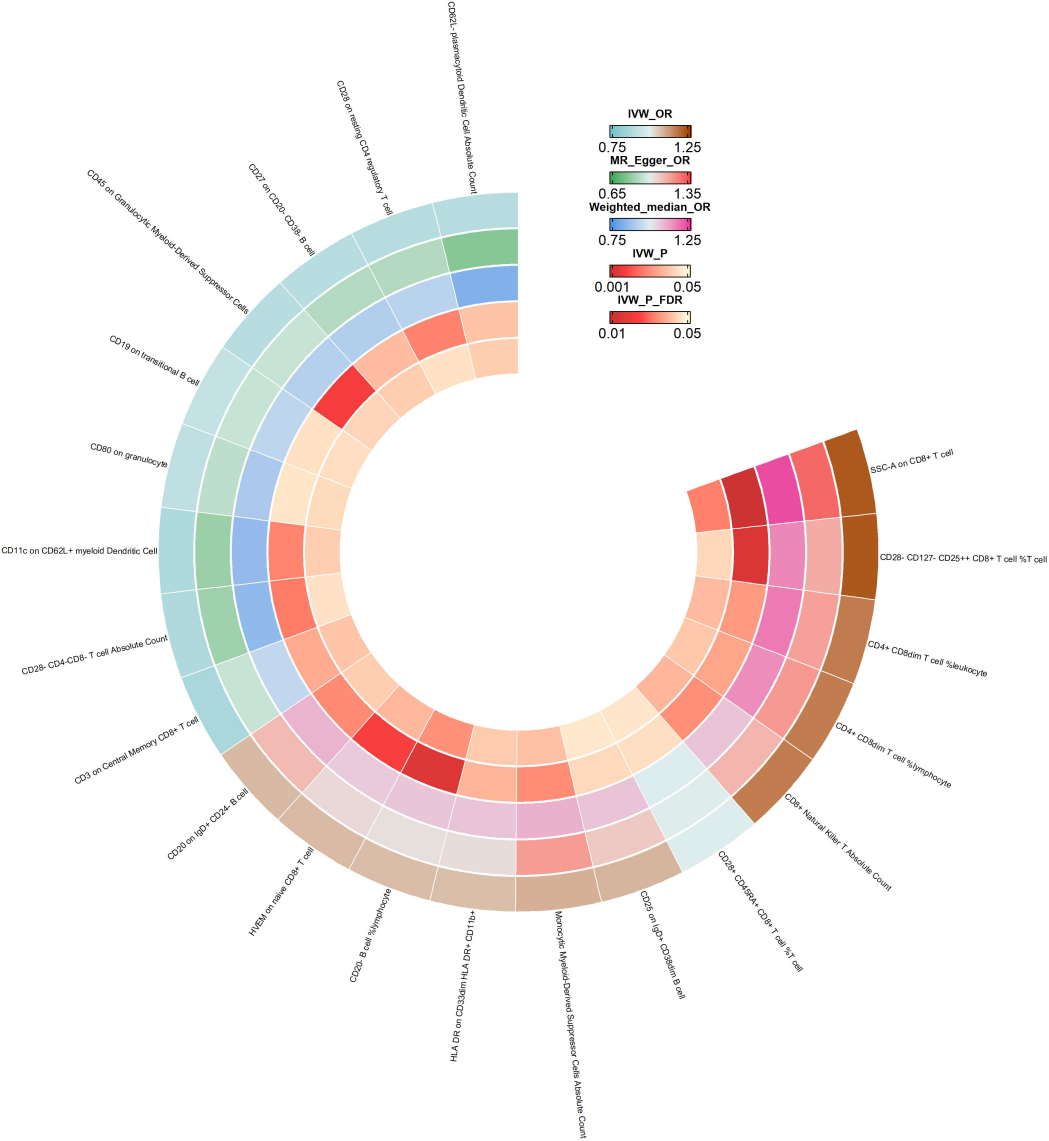
Ring heatmap of Mendelian randomization analyses of immune cell phenotype on esophageal cancer. IVW_OR, the results of odds ratio of inverse variance weighted method. MR_Egger_OR, the results of odds ratio of MR Egger method. Weighted_median_OR, the results of odds ratio of weighted median method. IVW_P, the P value for inverse variance weighted method. IVW_P_FDR, the P-Value after FDR adjust.

Our team conducted a thorough statistical analysis to explore the potential impact of immune cell phenotype on the development of esophageal cancer. Upon careful examination, we discovered that 27 immune cell phenotypes significantly affected esophageal cancer, as evidenced by a p-value of less than 0.05 (IVW) (for more information, please refer to **Supplementary Materials Table 3**). We then utilized the Weighted Median and MR Egger methods to eliminate exposure factors that had inconsistent directions of OR values across the three approaches. Through sensitivity analyses, we found no significant heterogeneity or level of pleiotropy. To enhance the reliability of our results, we applied FDR correction to the IVW results, which enabled us to identify exposure factors with a PVAL (FDR) >0.05. As a result of our efforts, we uncovered 21 immunophenotypes causally linked to esophageal cancer, and we created a circular heat map to visually represent our findings (see **Figure 3**).

### 3.4 Mediated MR analysis

**Figure 4:**
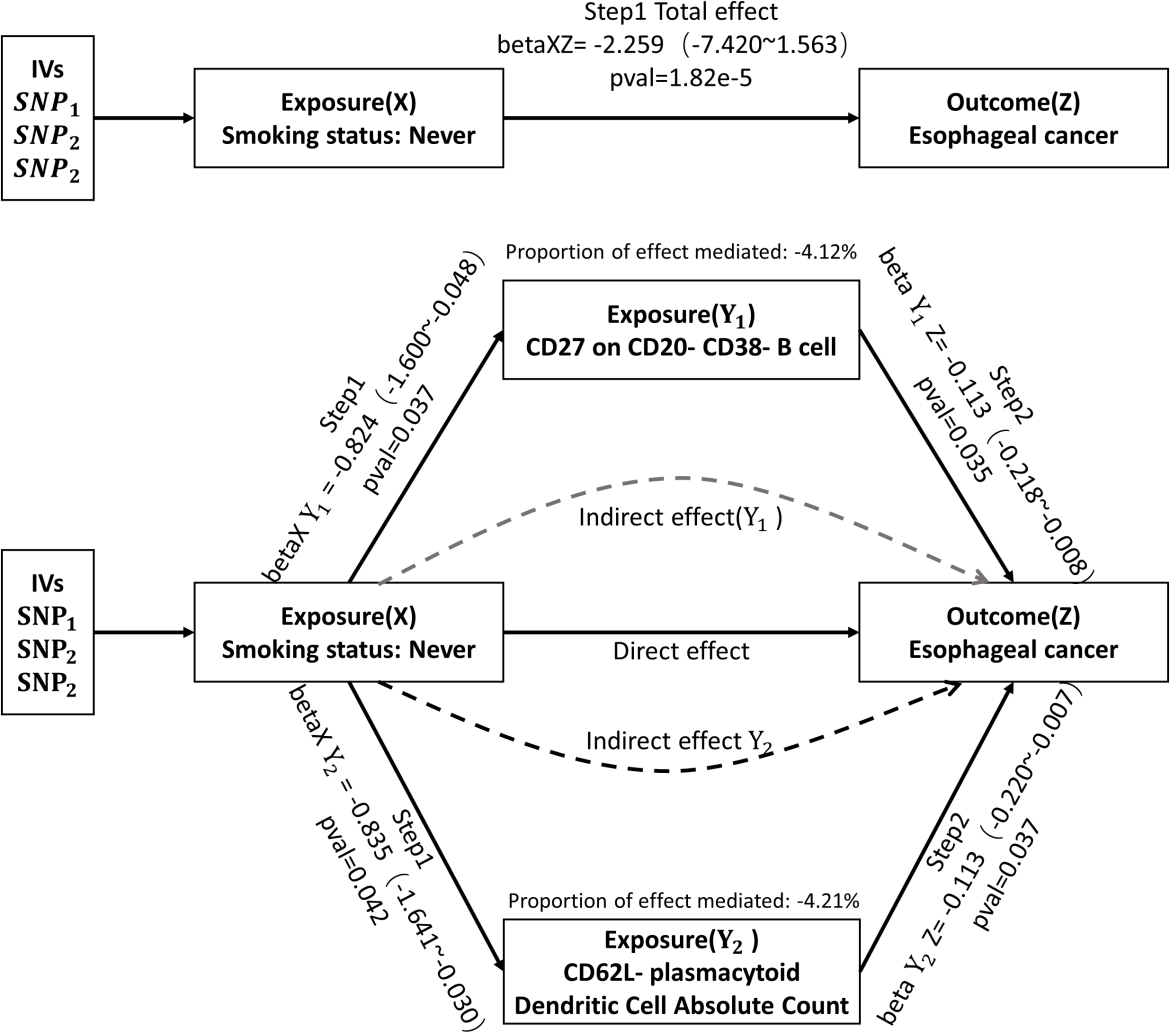
Mediation analysis of the effect of Smoking status: Never on esophageal cancer via immune cell phenotype. IVs, instrumental variables. SNP, single nucleotide polymorphisms.

To identify immunophenotypes that could be potential mediators, we explored the effect of Smoking status: Never on the 21 immunophenotypes derived from the above analysis. The results of all three assays and sensitivity analyses are presented in **Supplementary Materials Table 4**. After screening, two possible mediated immunophenotypes were obtained, including CD62L-plasmacytoid Dendritic Cell Absolute Count (IVW method, p=4.21e-2, OR=0.43, 95%CI=0.19~0.97) and CD27 on CD20-CD38- B cell (IVW method, p=3.73e-2, OR=0.44, 95%CI=0.20~0.95). We then explored the mediating effect of the above two immunophenotypes in the impact of Smoking status: Never on esophageal cancer. As shown in **Figure 4**, CD27 on CD20- CD38- B cell, as a potential mediator, reduced the intensity of Smoking status: Never in preventing or inhibiting esophageal cancer (mediating effect: -4.12%). Similarly, CD62L-plasmacytoid Dendritic Cell Absolute Count reduced the intensity of the negative impact of Smoking status: Never on the incidence of esophageal cancer (mediating effect: -4.21%).

## 4 Discussion

In this research, we employed genetic prediction to investigate how smoking-related traits impact the development of esophageal cancer. Furthermore, we explored immune cell phenotypes that may serve as mediators. Our results revealed that individuals who never smoked had a one-way causal relationship with a lower risk of esophageal cancer. We then conducted mediation analyses and ultimately identified two immunophenotypes that exhibited potential mediating effects: CD62L-plasmacytoid Dendritic Cell Absolute Count and CD27 on CD20- CD38- B cells. Using coefficient product analysis, we determined that both immunophenotypes mitigated the intensity of the effect of Smoking status: Never on the prevention or inhibition of esophageal cancer (mediating impact <0).

Esophageal cancer is a leading cause of cancer-related deaths worldwide, and multiple factors can contribute to its occurrence and progression (33). Numerous observational studies indicate that smoking is a significant risk factor for esophageal cancer. For instance, a large-scale prospective cohort study conducted in Japan found that heavy smoking (15 cigarettes/day or more) was significantly associated with increased mortality from esophageal cancer (RR=2.3, 95%CI=1.7~3.1) (34). Meanwhile, another retrospective study revealed that heavy smokers had a higher hazard ratio than non-heavy smokers (1.73, 95% CI: 1.12-2.68; P = 0.013) (35). Moreover, a meta-analysis of 41 studies on esophageal squamous cell carcinoma suggested that current smokers were four times more likely to develop this cancer type than non-smokers. However, prolonged smoking abstinence significantly decreased the likelihood of ESCC development (36). Although these studies suggest that smoking can contribute to the development and progression of esophageal cancer, they have certain limitations due to the lack of randomization, prospectivity, and blinding, which can lead to confounding interference. To address this issue, we utilized the Mendelian randomization (MR) method to analyze the causal relationship between smoking-related features and esophageal cancer. The instrumental variables (IV) in MR analysis are chosen as SNPs that are randomly distributed and not influenced by environmental or other exposure factors. Through our univariate two-way MR analysis, it was found that never smoking significantly lowers the risk of esophageal cancer. Conversely, the incidence of this cancer is promoted by ever-smoking and current tobacco smoking.

Smoking emits thousands of chemicals that can promote cancer by causing dysregulation and transformation of cells, as well as altering the immune microenvironment to favor cancer cell development and invasion (37, 38). Research conducted by Liu et al. suggests that smoking may activate mast cells and CD4 memory T cells, leading to tumor growth and progression (39). Additional studies have indicated that smoking alters innate and adaptive immunity in lung, breast, and colorectal cancers through the release of cytokines from cytotoxic or inflammatory cells (40–42). As a result, smoking could impact the occurrence and progression of esophageal cancer by influencing the host immune system. To account for individual smoking habits, we selected “Smoking status: Never” for a follow-up mediating study. Utilizing a two-step MR analysis, we identified two immune cell phenotypes - CD62L-plasmacytoid Dendritic Cell Absolute Count and CD27 on CD20- CD38- B cells - with potential mediating effects. Intriguingly, further analysis revealed that both immunophenotypes had the opposite effect, reducing the inhibitory effect of “Smoking status: Never” on esophageal cancer.

Plasmacytoid dendritic cells (pDCs) are a unique type of sentinel cell that detects pathogen-derived nucleic acids and responds rapidly by producing high volumes of type I interferons (43). L-selectin (CD62L) is a transmembrane glycoprotein and cell adhesion molecule found on the surface of several types of leukocytes (44). Although pDCs have demonstrated the potential to elicit anti-tumor immune responses, studies have shown that their infiltration in tumor microenvironments (TMEs) has varying effects on different cancers (44–47). Unfortunately, there is a lack of research on their direct role in esophageal cancer. In our study, we found that while CD62L-plasmacytoid Dendritic Cell Absolute Count had a negative causal effect on esophageal cancer, it weakened the preventive effect of never smoking on esophageal cancer. CD27, when it binds to its native ligand CD70, can promote T cell proliferation and differentiation into effector and memory T cells, which have potent anti-tumor potential. The CD27 agonist antibody varlilumab has shown promising efficacy in both hematologic and solid cancers (48). Similarly, our findings suggest that CD27 on CD20-CD38- B cells can inhibit the development of esophageal cancer. However, it’s worth noting that it has a negative mediating effect on the preventive effect of never smoking on esophageal cancer. One possible explanation for the negative mediating effect of the above immune cell phenotypes is that smoking may cause a partial immune response in the body, which may be lacking in never-smokers. It’s important to note that the specific mechanism of action requires further exploration by researchers.

Taken together, in this study, we genetically predicted the effect of smoking-related traits on esophageal cancer and further explored immune cell phenotypes with potential mediating effects. The results of our analysis showed that Smoking status: Never had a one-way causal relationship in the risk reduction of esophageal cancer, and based on this, subsequent mediation studies were conducted. Using a two-step MR analysis, we finally screened two immunophenotypes with potential mediating effects, namely CD62L-plasmacytoid Dendritic Cell Absolute Count and CD27 on CD20- CD38- B cell. After the coefficient product method, we found that both reduced the intensity of the effect of Smoking status: Never in preventing or inhibiting esophageal cancer (mediating effect<0)。The findings presented here provide additional evidence to substantiate the notion that the immune system plays a crucial mediating role in the association between environmental factors and cancer. The results underscore the importance of immune modulation as a preventive measure against cancer occurrence among high-risk populations. In addition, the phenotypic characteristics of the above two immune cells may be used as new indicators to predict the risk of esophageal cancer in high-risk groups such as smoking, which is conducive to the screening and prevention of esophageal cancer. At the same time, our study also provides a research method to explore the potential mediators of environmental factors and cancer associations.

This study presents several notable advantages. Firstly, we employed multiple complementary MR methods to explore the causal effects of smoking-related features and 731 immune cell phenotypes on esophageal cancer risk, minimizing the impact of residual confounders. Secondly, we delved into the mediating effect of immune factors on the risk of esophageal cancer, providing a valuable reference for future research into the mechanism of risk factors related to esophageal cancer. Finally, we conducted a comprehensive sensitivity analysis to ensure the reliability of our results.

However, there are some limitations to our research. On the one hand, overlapping participants in the samples of exposures and outcomes may impact the accuracy of MR analysis. To mitigate this, we utilized strong effect IV with F-statistic greater than 10 for all instrumental variables. On the other hand, our dataset only includes the European population, which may limit the generalizability of our findings to other people. While this approach reduces population stratification bias, it is essential to acknowledge that our results may not apply to other individuals.

## 5 Conclusion

In summary, our comprehensive MR analysis found that 3 smoking-related traits and 21 immune cell phenotypes had causal effects on esophageal cancer. On this basis, we further screened two immune cell phenotypes with potential mediating effects and calculated the intensity of the inhibitory effect of Smoking status: Never. This study provides new evidence for a causal relationship between smoking-related features and esophageal cancer and proposes immune factors with potential mediating effects. However, this finding needs to be further demonstrated by more extensive clinical studies.

## Data availability statement

The original contributions presented in the study are included in the article/**Supplementary Material**. Further inquiries can be directed to the corresponding author.

## Author contributions

KY: Data curation, Writing – original draft. SL: Data curation, Writing – original draft. YD: Visualization, Writing – original draft. XM: Software, Writing – original draft. CZ: Data curation, Writing – original draft. XS: Writing – review & editing.
